# Increase in α-tubulin modifications in the neuronal processes of hippocampal neurons in both kainic acid-induced epileptic seizure and Alzheimer’s disease

**DOI:** 10.1038/srep40205

**Published:** 2017-01-09

**Authors:** Hang Thi Vu, Hiroyasu Akatsu, Yoshio Hashizume, Mitsutoshi Setou, Koji Ikegami

**Affiliations:** 1 Department of Cellular and Molecular Anatomy, and International Mass Imaging Center, Hamamatsu University School of Medicine, Hamamatsu, Japan; 2 Choju Medical Institute, Fukushimura Hospital, Toyohashi, Japan; 3 Department of Medicine for Aging in Place and Community-Based Medical Education, Nagoya City University Graduate School of Medical Sciences, Nagoya, Japan; 4 Department of Systems Molecular Anatomy, Preeminent Medical Photonics Education and Research Center, Hamamatsu University School of Medicine, Hamamatsu, Japan; 5 Department of Anatomy, The University of Hong Kong, Hong Kong, China; 6 Division of Neural Systematics, National Institute for Physiological Sciences, Okazaki, Japan; 7 Riken Center for Molecular Imaging Science, Kobe, Japan

## Abstract

Neurodegeneration includes acute changes and slow-developing alterations, both of which partly involve common cellular machinery. During neurodegeneration, neuronal processes are impaired along with dysregulated post-translational modifications (PTMs) of cytoskeletal proteins. In neuronal processes, tubulin undergoes unique PTMs including a branched form of modification called glutamylation and loss of the C-terminal tyrosine residue and the penultimate glutamic acid residue forming Δ2-tubulin. Here, we investigated the state of two PTMs, glutamylation and Δ2 form, in both acute and slow-developing neurodegenerations, using a newly generated monoclonal antibody, DTE41, which had 2-fold higher affinity to glutamylated Δ2-tubulin, than to unmodified Δ2-tubulin. DTE41 recognised glutamylated Δ2-tubulin preferentially in immunostaining than in enzyme-linked immunosorbent assay and immunoblotting. In normal mouse brain, DTE41 stained molecular layer of the cerebellum as well as synapse-rich regions in pyramidal neurons of the cerebral cortex. In kainic acid-induced epileptic seizure, DTE41-labelled signals were increased in the hippocampal CA3 region, especially in the stratum lucidum. In the hippocampi of post-mortem patients with Alzheimer’s disease, intensities of DTE41 staining were increased in mossy fibres in the CA3 region as well as in apical dendrites of the pyramidal neurons. Our findings indicate that glutamylation on Δ2-tubulin is increased in both acute and slow-developing neurodegeneration.

Neurodegeneration includes a wide range of phenomena from acute changes to slow-developing alterations. An example of acute events is epileptic seizures, where neurons are damaged by excitotoxicity^1^. Slow-developing events include late-onset neurodegenerative diseases, such as Alzheimer’s disease (AD), in which neurons are gradually lost^2^. Despite the huge difference in the time span of neurodegeneration, both acute and slow-developing neurodegenerative pathways contain common cellular machinery and molecules. Several studies have revealed that dysregulated protein post-translational modifications (PTMs), including cytoskeletal proteins, are involved in neurodegeneration. In AD, a microtubule-associated protein, tau is hyperphosphorylated, which forms neurotoxic neurofibrillary tangles^3^. Currently believed mechanisms for tau aggregation involve self-aggregation of hyperphosphorylated tau and prion-like propagation of sequestering normal tau into the aggregates^4^. The aggregated tau is thought to be linked to impairments of neuronal function in AD by affecting microtubules stability and function as a ‘railway’ for neuronal transports^5^. Aberrant phosphorylation of neurofilaments is detected in a broad range of neurodegenerative diseases, including amyotrophic lateral sclerosis (ALS), AD, and Parkinson’s disease (PD)^6^. Dysregulation of SUMOylation is also reported in AD human brain and mouse AD model^7^.

Neurons have long and thin processes called neurites, or axons and dendrites. Neuronal processes are rich in microtubules composed of the building block, tubulin. In neurons, tubulin is subjected to a variety of PTMs in the C-terminal region, such as glutamylation^8^, detyrosination^9^,^10^, and conversion to untyrosinatable Δ2-tubulin^11^,^12^ and Δ3-tubulin^13^. Glutamylation is a highly unique form of PTM that generates a polyglutamate branch on a glutamic acid residue in the C-terminal region of tubulin^8^. The vast majority of neuronal tubulin undergoes glutamylation^14^. Tubulin glutamylation is, thus, important for maintaining neuronal function; for example, glutamylation of α-tubulin is essential for the KIF1-mediated transport of synaptic vesicle precursors to axonal terminals^15^.

Glutamylation is catalysed by a subfamily of the tubulin tyrosine ligase (TTL)-like (TTLL) protein family^16^,^17^,^18^. TTLL proteins possess a conserved core catalytic domain, TTL domain^16^. Eight TTLL proteins, TTLL1, 4, 5, 6, 7, 9, 11, and 13 are involved in tubulin glutamylation^16^,^17^,^18^,^19^. TTLL4 and 5 catalyse the first step, i.e., the initiation of glutamylation, with preferences to α- and β-tubulin, respectively^18^. TTLL5 also elongates the glutamate chain, i.e. “poly”-glutamylation, on α-tubulin^18^. TTLL6, 11, and 13 are involved in elongation of the glutamate chain on α-tubulin^18^. TTLL7 has a highly selective activity of both initiation and elongation on β-tubulin^18^,^20^. In neurons, α-tubulin polyglutamylation is mainly performed by TTLL1^21^, and β-tubulin polyglutamylation is catalysed by TTLL7^17^. Glutamylation is also reversed through deglutamylation by all members of cytosolic carboxypeptidases (CCPs)^22^,^23^. CCP1, 2, 3, 4, and 6 shorten polyglutamyl chains^23^,^24^,^25^,^26^. CCP5 has an additional function of removing a glutamate at the branching point by cutting the γ-α linkage^22^,^23^,^25^.

Detyrosination occurs through loss of the C-terminal tyrosine residue by unidentified carboxypeptidase(s)^27^, and detyrosinated tubulin is further converted to Δ2-tubulin through deglutamylation of the penultimate glutamic acid residue. The removal of penultimate glutamic acid residue is irreversible, resulting in the exclusion of Δ2-tubulin from the reversible cycle of detyrosination/retyrosination^28^. About 35% of neuronal α-tubulin is Δ2-tubulin^28^. The deglutamylation for generating Δ2-tubulin is catalysed by the same members of CCPs as the reverse reaction of polyglutamylation^23^,^24^,^25^,^26^. Δ3-tubulin is generated through the further removal of the third last glutamic acid residue from Δ2-tubulin by CCP1, 4, 5, and 6^24,13^.

Accumulating evidence also suggests that tubulin PTMs are involved in neurodegeneration. Dysregulated tubulin acetylation is reportedly associated with Huntington’s disease and PD^29^,^30^. Tubulin glutamylation is also involved in neurodegeneration. We and others have demonstrated that dysregulated over-glutamylation causes severe degeneration of cerebellar Purkinje cells in *Ccp1*-deficient mice^23^,^24^. The relative level of tubulin glutamylation against tubulin amount is increased in AD brain: signal of glutamylated tubulin is specifically increased in apical dendrites of the CA1 pyramidal neurons remaining in AD cases while the total amount of α-tubulin is decreased^31^. In addition, toxic amyloid-β (Aβ) transiently enhances tubulin glutamylation^32^.

For a better understanding of disease-related alterations in PTMs, precise and detailed analyses of PTMs are important. This is even more critical in the case where multiple PTMs occur on a molecule with each PTM located close to each other. Recently, detailed multi-phosphorylation states of tau have been analysed in AD brains using a new technique^33^. Despite long investigations on tubulin PTMs in the nervous system, few studies focus on the coexistence of multiple PTMs on an individual tubulin molecule in neurodegenerative conditions as well as in the normal brain. In this study, we generated a monoclonal antibody (mAb), DTE41 that had better affinities to the two modifications, glutamylation and conversion to Δ2 form on individual α-tubulin molecules, which we called glutamylated Δ2-tubulin. We found that DTE41-detected signals were increased in the stratum lucidum of the hippocampal CA3 region in kainic acid-administered mouse brain and in the hippocampus, especially mossy fibres of the CA3 region, in post-mortem AD brains.

## Results

### Characterisation of a novel monoclonal antibody, DTE41, targeting glutamylated Δ2 form of α-tubulin

**Figure 1:**
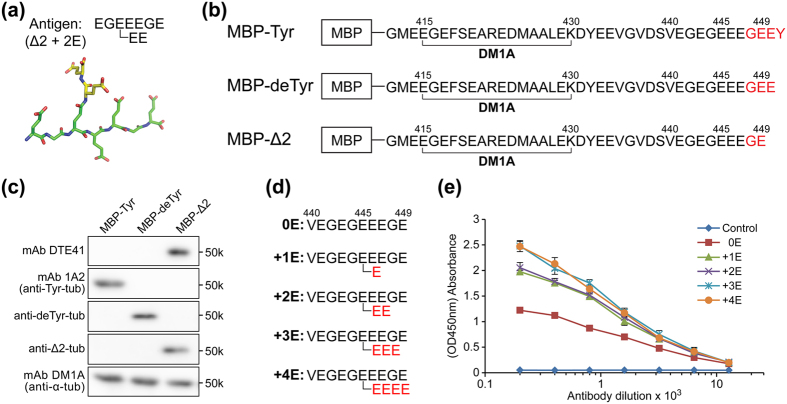
Characterisation of a novel monoclonal antibody, DTE41, targeting glutamylated Δ2 form of α-tubulin. (**a**) Antigen used to generate mAb DTE41. The C-terminal part of Δ2 form with di-glutamylation was used. Colour codes of the rod structure of antigen peptide are green, carbohydrate chain in Δ2 form; yellow, carbohydrate chain of branched di-glutamates; blue, nitrogen; red, oxide. The peptide has in total 8 carboxyl groups. (**b**) Schematic view of recombinant α-tubulin tail fused to maltose binding protein (MBP) with different C-terminal modifications, tyrosination (Tyr), detyrosination (deTyr), and Δ2 form. Epitope of DM1A is indicated. (**c**) Preference of mAb DTE41 to Δ2-tubulin. Recombinant α-tubulin C-termini with different modifications were subjected to immunoblotting. Original full-length blot images were provided in Supplementary Figure 6a. (**d**) Schematic view of peptides used for ELISA. (**e**) ELISA of mAb DTE41 against peptides shown in panel **d**.

We generated a mAb, named DTE41, to target a short peptide of C-terminal region of Δ2-tubulin undergoing di-glutamylation (Fig. 1a). The primary and deduced quaternary structures of the variable region of mAb DTE41 showed 9 basic amino acids on the surface of the antigen-recognising domain (Supplementary information; Fig. S1a, S1b, and S1c). We first confirmed that mAb DTE41 detected preferentially Δ2 form of α-tubulin by using recombinant tubulin tails having 3 different C-terminals, tyrosinated, detyrosinated, and Δ2 forms, which were fused to maltose-binding protein (MBP) (Fig. 1b). mAb DTE41 specifically recognised the Δ2 form of the α-tubulin tail while failing to react to tyrosinated or detyrosinated forms (Fig. 1c and S2a).

We next examined whether DTE41 reacted preferentially to the glutamylated form of Δ2-tubulin with indirect enzyme-linked immunosorbent assay (ELISA). Synthetic peptides with different numbers of glutamyl units from 0–4 were used for the analysis (Fig. 1d). mAb DTE41 reacted ~2-fold effectively to glutamylated Δ2-tubulin than to unmodified Δ2-tubulin (Fig. 1e). Of note, the reaction efficiency of the mAb to glutamylated Δ2-tubulin was increased upon the increase in the number of glutamates in the branch (Fig. 1e). Anti-Δ2-tubulin polyclonal antibody (pAb) showed no dependency of Δ2 form recognition on glutamylation (Fig. S2a). mAb DTE41 did not recognise only-glutamylated tubulin, i.e. glutamylated Tyr-tubulin (Fig. S2b). Taken together, these results demonstrate that mAb DTE41 preferentially recognises highly glutamylated Δ2-tubulin.

### mAb DTE41 preferentially recognises glutamylated Δ2-tubulin-enriched microtubules in cultured cells

**Figure 2:**
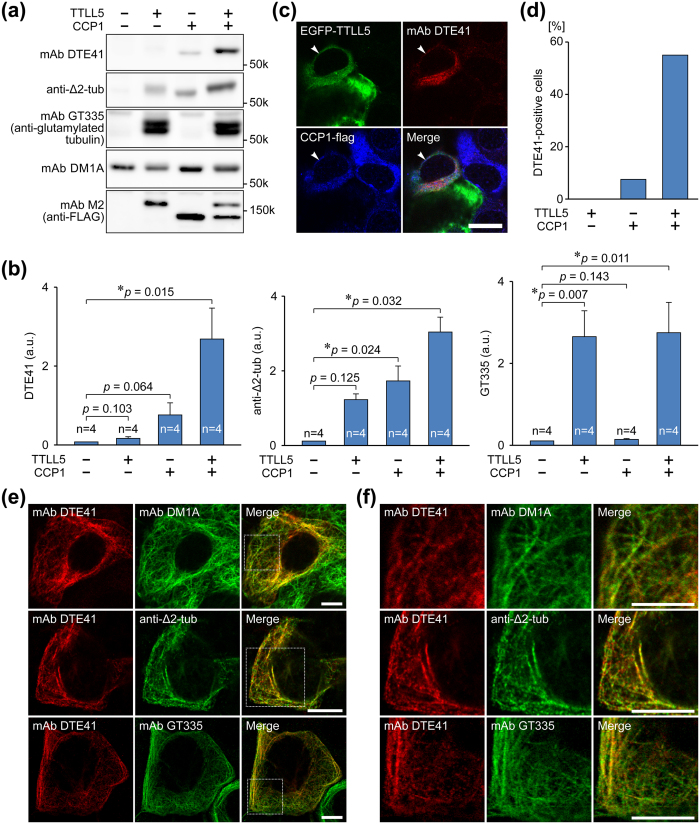
mAb DTE41 preferentially recognises microtubule enriched by the glutamylated Δ2 form in cultured cells. (**a**) Representative western blot analyses with 3 different antibodies in HEK293T cells. Original full-length blot images were provided in Supplementary Figure 6b. (**b**) Quantitative analyses of western blotting as shown in panel **a**. Results are shown as the mean of four independent experiments. Error bars represent S.E.M. (**c**) Immunocytochemical analyses of mAb DTE41 in COS7 cells. mAb DTE41 was reactive only to cells expressing both TTLL5 and CCP1 (arrowhead). Scale bar, 20 μm. Green, EGFP-TTLL5; red, DTE41; blue, CCP1-flag. (**d**) Quantification of mAb DTE41-positive cells in panel **c**. (**e,f**) Co-labelling of modified microtubule with mAb DTE41, mAb GT335, and anti-Δ2-tubulin pAb. COS7 cells or IMCD3 cells co-expressing TTLL5 and CCP1 were labelled with mAb DTE41 and mAb DM1A, mAb GT335, or anti-Δ2-tubulin pAb. Red, DTE41; green, DM1A (upper), Δ2-tubulin (middle), or GT335 (bottom); blue, DAPI. Panel **f** shows magnified images in a box of dashed line in panel **e**. Scale bar, 10 μm.

We tested whether mAb DTE41 preferentially detected glutamylated Δ2-tubulin in the cells expressing a glutamylating enzyme, TTLL5, and a Δ2 form-generating enzyme, CCP1. mAb DTE41 showed the strongest reactivity to a 50-kDa protein in the cells co-expressing both CCP1 and TTLL5 (Fig. 2a) with a statistically significant efficiency (Fig. 2b). It also reacted weakly to a 50-kDa protein in the cells expressing only CCP1 (Fig. 2a), though the statistical value was more than 0.05 (Fig. 2b). The conversion of α-tubulin to the Δ2 form by CCP1 expression was verified with a specific anti-Δ2 polyclonal antibody (Fig. 2a and b). Glutamylation of α-tubulin by TTLL5 was also confirmed with mAb GT335, a specific mAb against glutamylated tubulin (Fig. 2a and b). Upper-shifting of tubulin bands detected with mAb DTE41 or anti-Δ2-tubulin pAb were observed in TTLL5-overexpressing samples, while DM1A-detected tubulin bands did not show such band-shifting (Fig. 2a), indicating that only a part of tubulin in cells was glutamylated. A surprising result was that anti-Δ2-tubulin pAb showed an increase of band intensity in cells expressing TTLL5 alone (Fig. 2a and b).

We next tested whether mAb DTE41 detected modified proteins using immunocytochemistry on COS7 cells co-expressing TTLL5 and CCP1. mAb DTE41 signal was detected only in cytoplasm of cells that expressed both TTLL5 and CCP1 (Fig. 2c; arrowhead). The antibody did not detect any signals in cells expressing either TTLL5 or CCP1 (Fig. 2c). To evaluate the detection efficiency and selectivity of mAb DTE41 against glutamylated Δ2-tubulin, we quantified the DTE41-positive cells expressing TTLL5 alone, CCP1 alone, or both TTLL5 and CCP1. mAb DTE41 recognised about 50% of cells expressing both TTLL5 and CCP1 (Fig. 2d). In contrast, it detected only a few or none of the cells expressing CCP1 alone or TTLL5 alone, respectively (Fig. 2d), indicating that mAb DTE41 had better affinity to glutamylated Δ2-tubulin in immunostaining compared to ELISA and immunoblots.

We next confirmed whether the cytoplasmic signal detected by mAb DTE41 was tubulin. The DTE41-labelled signals in the cytoplasm were overlapped with microtubules visualised with DM1A (Fig. 2e and f). We further examined whether mAb DTE41-labelled structures are also co-localised with glutamylated tubulin and Δ2-tubulin. The DTE41 signals were co-localised with both signals of anti-Δ2-tubulin pAb and mAb GT335 (Fig. 2e and f). Note that some cells labelled with anti-Δ2-tubulin pAb were negative for mAb DTE41 staining (Fig. S2c), supporting the idea that mAb DTE41 detects microtubules that possess glutamylated Δ2-tubulin preferentially in immunostaining than in immunoblotting.

### mAb DTE41 stained soma and dendrites of hippocampal and cortical pyramidal neurons and distal dendrites of cerebellar Purkinje neurons

**Figure 3:**
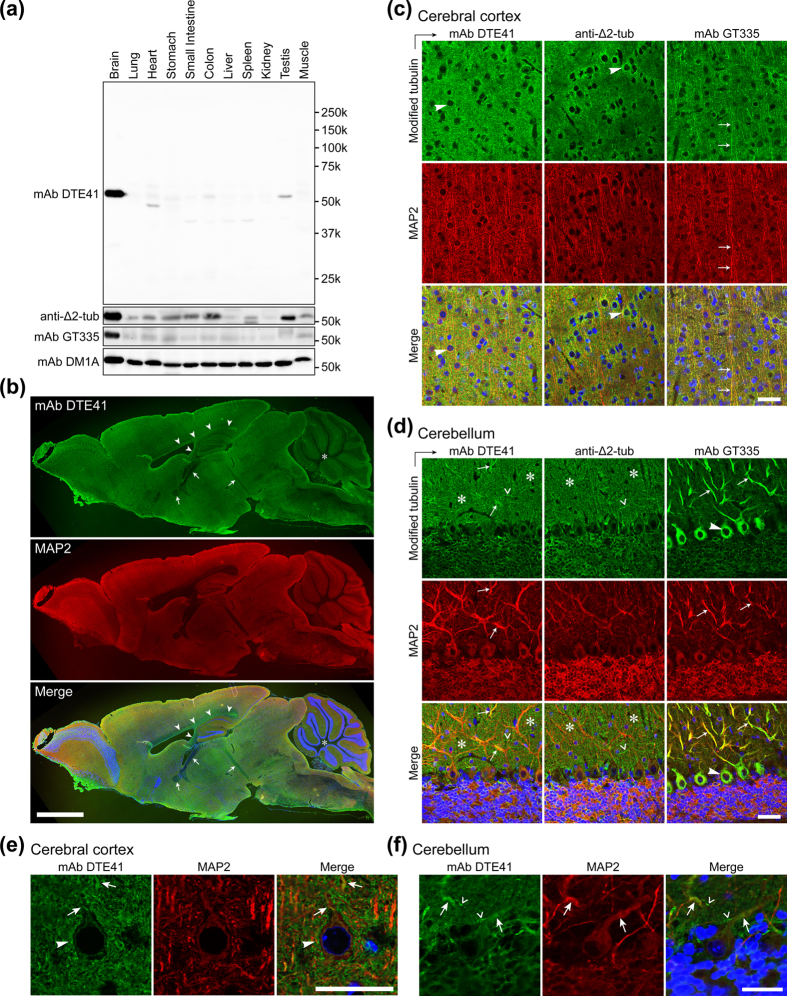
mAb DTE41-labelled mouse brain neurons showed a similar staining pattern to that of anti-Δ2-tubulin pAb and different from mAb GT335. (**a**) Representative western blot analyses of mouse tissue extracts with mAb DTE41, mAb GT335, and anti-Δ2-tubulin pAb. Original full-length blot images of GT335, anti-Δ-2-tubulin pAb, and mAb DM1A were provided in Supplementary Figure 6c. (**b**) Immunohistochemical staining of whole mouse brain section with mAb DTE41 and MAP2. Arrowheads: corpus callosum, and ventral hippocampal commissure. Arrows: the anterior commissure, fornix, and thalamic stria medullaris. Asterisk, white matter of the cerebellum. Green, DTE41; red, MAP2; blue, nuclei. Scale bar, 2 mm. (**c,d**) Immunohistochemical staining of tissue sections of the cerebral cortex (**c**) and cerebellum (**d**) with mAb DTE41, mAb GT335, and anti-Δ2-tubulin pAb. Arrowhead, cell body; arrow, dendrite; asterisk, substrate of molecular layer of the cerebellum; check mark, climbing fibre. Green, modified tubulins; red, MAP2; blue, nuclei. Scale bar, 40 μm. (**e,f**) Magnified images of DTE41 staining on the cerebral cortex (**e**) and cerebellum (**f**). Scale bar, 30 μm. Green, DTE41; red, MAP2; blue, nuclei.

We tested the expression level of DTE41-detected tubulin in the nervous system with immunoblot and immunohistochemical analyses. The immunoblot analysis with mAb DTE41 showed that the antibody target was highly abundant in the mouse brain lysate (Fig. 3a). Other organs contained none of the double-modified form, or only faint level if present, as in the testis (Fig. 3a). In contrast, Δ2-tubulin was detected not only in the brain but also in many other organs including heart, stomach, small intestine, colon, testis, and muscle (Fig. 3a). Glutamylation was detected selectively only in the brain (Fig. 3a).

We next investigated the distribution of DTE41-detected tubulin in mouse brain by immunohistochemistry. DTE41-detected tubulin was detected throughout the brain (Fig. 3b). Neither non-specific binding of secondary anti-mouse antibodies to residual mouse immunoglobulins nor cross-reactivity of mAb DTE41 to targets other than glutamylated Δ2-tubulin were detected in DTE41-stained samples (Fig. S3a and S3b). mAb DTE41 stained the internal granule cell layer of the olfactory bulb, the frontal cortex, the molecular layer (layer I) of the cerebral cortex, the thalamus, and the molecular layer of the cerebellum (Fig. 3b). Of interest, mAb DTE41 signal was absent in axon-rich regions, such as the anterior commissure, the fornix, the thalamic stria medullaris, the white matter of cerebellum, and neural tracts of the medulla oblongata, except for the corpus callosum and the ventral hippocampal commissure (Fig. 3b). The DTE41-detected substrates were almost overlapped with Δ2-tubulin, with minor differences such as stronger mAb DTE41 signals in the corpus callosum (Fig. S4a). We further analysed in more detail three different regions of the mouse brain, cerebral cortex, cerebellar cortex, and hippocampus.

In the cerebral cortex, DTE41-detected signals were predominantly localised in the periphery of cell bodies of pyramidal neurons (Fig. 3c, left, arrowhead), whereas limited colocalisation was observed with MAP2-positive dendrites. Highly-magnified photomicrographs showed that mAb DTE41 stained granular substrates in the cerebral cortex (Fig. 3e), indicating that they were localised in regions close to the synapse. DTE41 signals were also strongly detected on the surface of the cell body (Fig. 3e, arrowhead). MAP2-positive structures were infrequently detected by mAb DTE41 (Fig. 3e, arrow). The distribution of DTE41-detected signals in the cerebral cortex was highly similar to that of the Δ2-tubulin, which was also abundant in the periphery of cell bodies and negative in MAP2-positive dendrites (Fig. 3c, middle). Double staining with mAb DTE41 and anti-Δ2-tubulin pAb further demonstrated that signals detected with the two antibodies were almost overlapped (Fig. S4b). These results were in contrast to the distribution of GT335-detected glutamylated tubulin, which overlapped with MAP2-postive dendrites (Fig. 3c, right, arrow).

In the cerebellum, DTE41-detected signals were most strongly detected in the molecular layer (Fig. 3d, left, asterisks), localising in spots in the MAP2-positive main dendritic shaft of Purkinje neurons (Fig. 3d, left, arrow). In addition, DTE41-detected signals were detected in thin climbing fibres around soma and dendrites of Purkinje neurons (Fig. 3d, left, check marks). The cell bodies of Purkinje neurons were only weakly stained by mAb DTE41 (Fig. 3d, left). Highly-magnified photomicrographs showed that mAb DTE41 stained granular substrates in the molecular layer (Fig. 3f), indicating that they were localised close to synapses. Figure 3f also shows that DTE41 signals were detected in a portion of MAP2-positive main dendritic shafts of Purkinje neurons (Fig. 3f, arrow), and thin climbing fibres (Fig. 3f, check mark). Δ2-tubulin was also detected in the molecular layer (Fig. 3e, middle, asterisks), but absent in MAP2-positive dendrites of Purkinje neurons (Fig. 3e, middle). Similar to DTE41-detected signals, Δ2-tubulin was also detected in climbing fibres (Fig. 3e, middle, check mark) but not in the cell bodies of Purkinje neurons (Fig. 3e, middle). Similar staining patterns of mAb DTE41 and anti-Δ2-tubulin pAb were also observed in double staining (Fig. S4c). Glutamylated tubulin was highly abundant in the cell bodies and MAP2-positive main shaft of dendrites of Purkinje neurons (Fig. 3d, right, arrow and arrowhead).

In the hippocampus, DTE41-labelled signals were detected in the cell bodies of pyramidal neurons (Fig. S4d, left), and also present as puncta in the regions of apical dendrites of pyramidal neurons (Fig. S4d, left). Double staining with mAb DTE41 and anti-Δ2-tubulin pAb showed that signals detected with the two antibodies were mostly overlapping (Fig. S4d).

### DTE41-detected tubulin is increased in the stratum lucidum of the hippocampal CA3 region under acute kainate-induced epileptic seizure

**Figure 4:**
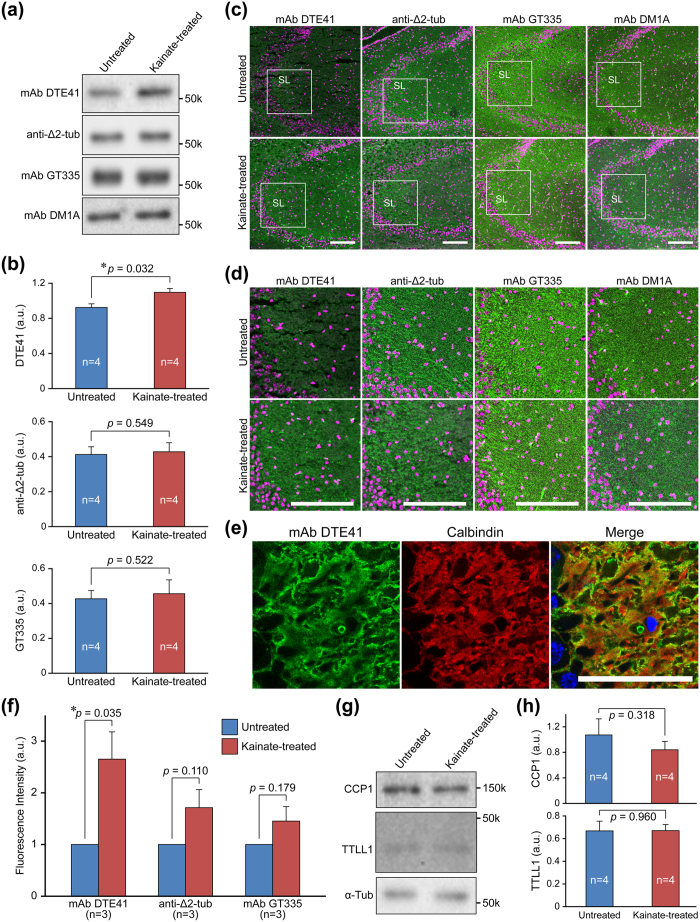
mAb DTE41 immunoreactivity is increased in the mossy fibre-rich layer of the hippocampal CA3 region in kainate-induced epilepsy. (**a**) Representative western blots of mAb DTE41, mAb GT335, and Δ2-tubulin in the hippocampus of kainate-treated mouse. Original full-length blot images were provided in Supplementary Figure 7a. (**b**) Quantitative analyses of western blotting as shown in panel **a**. Results are shown as the mean of 4 independent experiments. Error bars represent S.E.M. (**c**) Immunohistochemical staining of the hippocampal CA3 region with mAb DTE41. SL, stratum lucidum. Scale bar, 100 μm. (**d**) Magnified photographs in the square of the panel **c**. Scale bar, 100 μm. (**e**) Co-staining of the kainate-administrated hippocampal CA3 region with mAb DTE41 and anti-calbindin pAb. (**f**) Quantitative analyses of immunofluorescence signal in panel **d**. Results are shown as the mean of 3 independent experiments. Error bars represent S.E.M. (**g**) Representative western blots of TTLL1 and CCP1 in the hippocampus of kainate-treated mouse. Original full-length blot images were provided in Supplementary Figure 7b. (**h**) Quantitative analyses of western blotting shown in panel **g**. Results are shown as the mean of 4 independent experiments. Error bars represent S.E.M.

We examined whether the level of DTE41-detected tubulin was altered during an acute phase of neuronal degeneration. To this end, we analysed the modification level by DTE41 immunoblotting in the hippocampi of kainate-induced epileptic seizure mouse model. The DTE41-detected signal was significantly increased in the kainate-treated hippocampus compared to untreated controls (Fig. 4a and b). In contrast, the glutamylation and Δ2-tubulin signal were not changed (Fig. 4a and b).

We next confirmed the increase in DTE41-detected signals in the hippocampus through immunohistochemical staining. We focused on the hippocampal CA3 region since the region was most affected by kainate administration. DTE41-detected tubulin was increased in the hippocampal CA3 region by kainate administration (Fig. 4c, left), particularly in the stratum lucidum (Fig. 4d, left). In contrast, Δ2-tubulin and glutamylated tubulin appeared to be unaltered or barely increased in the CA3 region and the stratum lucidum (Fig. 4c and d). The mAb DTE41-labelled signals were not non-specific detections of leaked or residual immunoglobulins (Fig. S5a). The increase in DTE41 signals in the stratum lucidum of the CA3 regions was reproduced in kainate-administrated rats (Fig. S5b and S5c). Higher magnification demonstrated that signals detected with mAb DTE41 were localised on mossy fibres, which were positive for calbindin (Fig. 4e). In particular, DTE41 signals were strongest at the periphery of calbindin signals, indicating that the targets of mAb DTE41 were localised near synapses in mossy fibres (Fig. 4e).

Quantitative analyses of immunofluorescence intensities in the CA3 region showed that DTE41-detected signals were significantly increased by 2.5-fold compared to the control in the kainate-administered hippocampal CA3 region (Fig. 4f). The level of Δ2-tubulin showed a slight tendency of increase after the kainate administration; however, there was no statistical significance (Fig. 4f). The change of glutamylated tubulin level was not statistically significant in the kainate-administered versus the control CA3 region (Fig. 4f). The levels of two enzymes, TTLL1 and CCP1, which are predominantly involved in regulation of tubulin glutamylation and Δ2-tubulin formation in the brain, were not changed in the kainate-treated hippocampus (Fig. 4g and h). Our data demonstrated that DTE41-detected tubulin was increased in the acute phase of neurodegeneration.

### DTE41-detected tubulin is increased in the apical dendrites of pyramidal neurons and mossy fibres of the CA3 hippocampal region in Alzheimer’s disease (AD)

**Figure 5:**
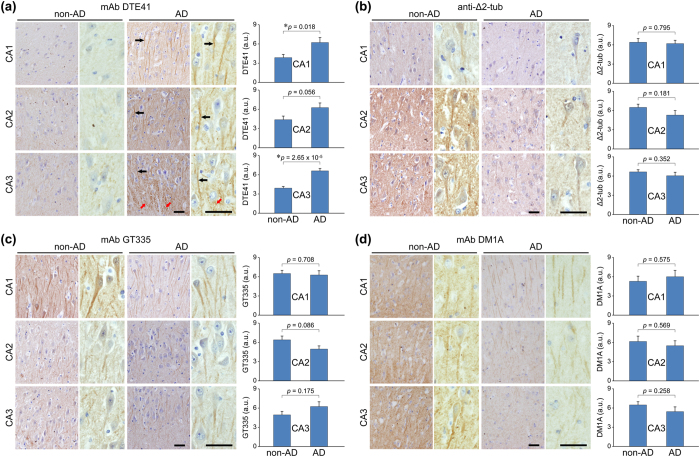
mAb DTE41 immunoreactivity is increased in the hippocampus, especially in mossy fibres of the CA3 region in Alzheimer’s disease brain. (**a**) Immunohistochemical staining of Alzheimer’s disease (AD) brain and non-AD brain with mAb DTE41. Black arrows, apical dendrites; red arrows, mossy fibres. Graphs show the quantified DTE41-labled signal in each region of the hippocampus. Error bars represent S.E.M. (**b**–**d**) Immunohistochemical staining of AD brain and non-AD brain with mAb GT335 (**b**), anti-Δ2-tubulin pAb (**c**), and mAb DM1A (**d**). In each data set, left are lower magnified images, and right are higher magnified images. Scale bars, 50 μm in all photomicrographs. Brown pigments are signals stained with antibodies. Graphs show the quantified signal intensities of each antibody in each hippocampal region.

**Figure 6:**
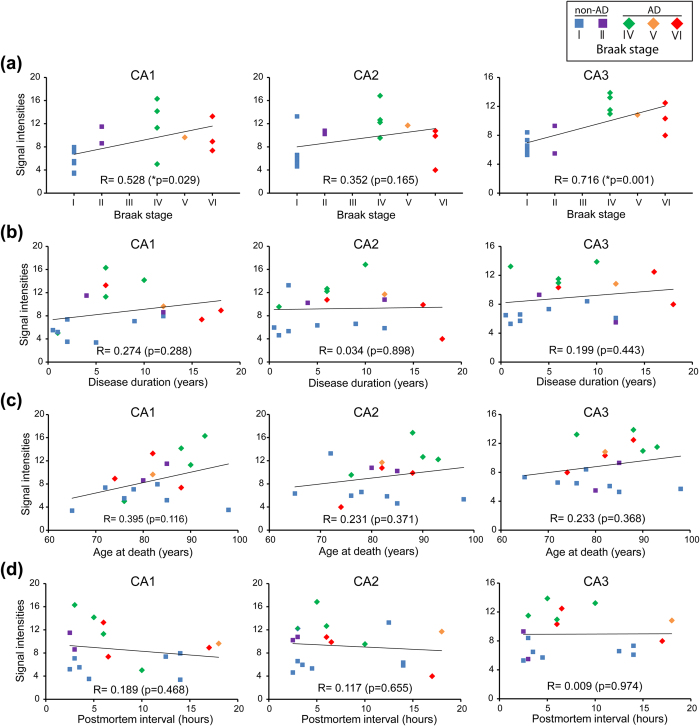
Correlation analyses of mAb DTE41-detected signal intensities against AD Braak stage, duration of disease, age at death, and postmortem interval. (**a–d**) The levels of DTE41-labelled signals plotted against Braak stages (**a**), disease duration (**b**), age at death (**c**), and post-mortem interval (**d**). A Pearson’s test were performed to evaluate the correlations between the parameters.

Next, we examined whether DTE41-detected tubulin was altered in a slow-developing neurodegenerative disease, AD, by immunostaining post-mortem brain specimens. We analysed the hippocampus of 8 AD brain samples and 9 non-AD brain samples (Table 1). In all the CA1, CA2, and CA3 regions of AD hippocampus, DTE41-detected signals were significantly increased (Fig. 5a, right graph). In particular, the signals were prominent in apical dendrites of the hippocampal pyramidal neurons of AD brain (Fig. 5a, black arrows). Of note, the DTE41-detected signals were remarkably increased in mossy fibres of the CA3 region in AD hippocampus (Fig. 5a, red arrows). The level of Δ2-tubulin, in contrast, showed a slight tendency of decrease in AD hippocampi compared to non-AD controls, though the difference was not statistically significant (Fig. 5b, right graphs). Glutamylated tubulin was detected in the apical dendrites of pyramidal neurons in all the hippocampal CA1, CA2 and CA3 regions at comparable levels in both AD and non-AD samples (Fig. 5c), with a tendency of decrease in the CA2 region and increase in the CA3 region of AD samples (Fig. 5c, right graph). α-Tubulin level detected with mAb DM1A showed marginal difference between the non-AD and AD hippocampi (Fig. 5d). The level of DTE41-detected signals was correlated only with the Braak stage (Fig. 6a). Of interest, Fig. 6a shows that the highest signals tend to be at the Braak stage IV. The disease duration, age at death, and post-mortem interval were not correlated to the level of DTE41-stained signals (Fig. 6b,c and d). Our data demonstrate that DTE41-detected tubulin was increased in AD, a slow-developing neurodegenerative disease.

## Discussion

In this study, we generated mAb DTE41 that targeted to glutamylated Δ2-tubulin. mAb DTE41 recognised ‘naked’ Δ2-tubulin as well as glutamylated Δ2-tubulin with about two-fold higher affinity on the “double-modified” tubulin (Fig. 1). This result indicates that mAb DTE41 recognises primarily Δ2-tubulin and glutamylated Δ2-tubulin in addition. mAb DTE41 did not recognise glutamylated Tyr-tubulin (Fig. S2b). This double dependency on a poly-glutamate branch and C-terminal states is different from the characteristic of mAb ID5: its interaction to α-tubulin depends on the –EE motif, which is common for both detyrosinated form and glutamylation^34^. This double dependency is in contrast to other commonly used mAbs for glutamylated tubulin, GT335 and B3. Both GT335 and B3 are thought to recognise glutamylation regardless of C-terminal states^35^. Given that Δ2-tubulin purified from bovine brain also undergoes polyglycylation^36^, the effects of polyglycylation on the recognition efficiency of DTE41 for Δ2-tubulin are of interest for future studies.

One could argue that mAb DTE41 simply detects Δ2-tubulin. This might be true as signals detected with mAb DTE41 and anti-Δ2-tubulin pAb mostly overlapped in the whole brain section (Fig. S4). However, a possibility remains that mAb DTE41 detected glutamylated Δ2-tubulin in some situations. Our data indicate that one such situation occurs in immunostaining. In immunocytochemistry, for instance, cells labelled with anti-Δ2-tubulin pAb were not necessarily positive for mAb DTE41 (Fig. S2c). This supports the notion that DTE41 recognises the additional glutamylation on Δ2-tubulin preferentially in immunostaining.

The initial evaluations of mAb DTE41 revealed two unexpected results. One is that DTE41 preferentially recognised the Δ2-tubulin C-terminal parts that had longer glutamate chains than those carrying shorter chains, although the antibody was generated against di-glutamylated Δ2-tubulin. This could result from the presence of 9 basic amino acids on the surface of antigen recognition pocket of DTE41 (Fig. S1), which is incidentally equal to the number of glutamic acid residues on the quad-glutamylated Δ2-tubulin C-terminal portion. It is not surprising that a mAb shows different target preferences in immunoblot and immunostaining. The other unexpected result is that TTLL5 induced an increase in Δ2-tubulin expression in HEK293T cells. A possible mechanism is that TTLL5-mediated glutamylation stabilises microtubules, which results in the Δ2-tubulin increase^37^. Our data excluded the possibility that the anti-Δ2-tubulin pAb used in this study recognises glutamylation on Δ2-tubulin as the pAb reacted to Δ2-tubulin independent of glutamylation. One would expect that mAb DTE41 could detect the possible Δ2-tubulin generated by TTLL5 overexpression. However, mAb DTE41 failed to detect tubulin in TTLL5-overexpressing samples (Fig. 2b and d). One explanation might be that mAb DTE41 could not bind to Δ2-tubulin tail with long poly-glutamate chains. Indeed, mAb DTE41 did not detect highly acidic α-tubulin with long polyglutamate chain (Fig. S2b). mAb DTE41 might have an optimal range of glutamate residues to recognise Δ2-tubulin. The length of polyglutamate chain on Δ2-tubulin might fall in the optimal range for DTE41 binding by the co-expression of CCP1 with TTLL5.

DTE41-detected signals were abundantly detected in the neurons of mouse brain (Fig. 3). This result is expected since the vast majority of tubulin is glutamylated in the brain^14^ and about 35% of tubulin is Δ2 form^28^. In addition, both modifications are accumulated in highly stable microtubules^12^,^38^. In fact, glutamylated Δ2-tubulin is higher in the adult brain, where neuronal processes and their microtubules are more stable than that in neonatal brain^39^. It is plausible that tubulin detected with mAb DTE41 in mouse brain under the normal conditions is predominantly Δ2-tubulin since the staining pattern of mAb DTE41 largely overlapped with Δ2-tubulin (Fig. S4). Another explanation, however, could be possible that Δ2-tubulin and glutamylated Δ2-tubulin happened to show almost identical distribution. This is likely, given that the vast majority of tubulin is glutamylated in the brain^14^, and ‘naked’ Δ2-tubulin was hardly detected in the brain^15^. In the cerebellum, the distribution of mAb DTE41 signals was almost identical to that of Δ2-tubulin (Fig. S4c)^12^. In contrast, the difference between mAb DTE41 and mAb GT335 staining patterns was prominent in the cerebellum, while that in the cerebral cortex was subtle. In the soma and the main shaft of Purkinje neurons, glutamylated tubulin detected by GT335 is likely to be mostly tyrosinated or detyrosinated forms. Indeed, both glutamylated tubulin and tyrosinated tubulin have been strongly detected in soma and the main shaft of Purkinje neurons^40^. In contrast, parallel fibres and climbing fibres have some populations of Δ2-tubulin that seem to undergo glutamylation with glutamate chains of optimal length for DTE41. In particular, the granular staining of DTE41 and Δ2-tubulin in the molecular layer of cerebellum and cerebral cortex suggests that Δ2-tubulin and glutamylated Δ2-tubulin were abundant near the axon terminals close to synapses.

DTE41-detected signals were increased upon acute epileptic seizure induced by kainic acid administration (Fig. 4). The increase of DTE41-detected signals was clear in immunohistochemistry, while being modest in western blotting. A possible reason of the difference is that tubulin extracted from CA1 and CA2 regions could diminish the increase of DTE41 signal in CA3 region. Another explanation is that DTE41 recognises the glutamylation on Δ2-tubulin more preferentially in immunohistochemistry than in western blotting, as discussed above for the results of Fig. 2. Given that Δ2-tubulin showed marginal increase in response to kainic acid administration, the increase in DTE41 signals could reflect an increase in glutamylated Δ2-tubulin. In this scenario, Δ2-tubulin might preferentially undergo glutamylation with longer glutamate chains, and/or glutamylated tubulin with longer glutamate chains might be converted to Δ2-tubulin. This raises a question whether glutamylated tubulin or Δ2-tubulin undergoes another modification. Some evidence implies that both modifications are altered simultaneously. A key report provides evidence that tubulin glutamylation is increased by neuronal activity in the central nervous system^41^. Given that kainic acid causes hyperexcitation of neuronal cells^42^, enhanced neuronal activation could induce the increase in glutamylated Δ2-tubulin. Enzymatic activity seems to be modulated, since the total amount of enzyme did not increase (Fig. 4g and h). A key molecule of kainic acid-mediated excitotoxicity is calcium ion^43^. *In vitro* enzymatic activity of CCP1, a Δ2-tubulin-generating enzyme, prefers the presence of calcium ion (Ca^2+^)^24^. Ca^2+^ increases the activity of CCP1 enzyme that leads to the increased signal of Δ2-tubulin. The region where DTE41-detected signals were increased is consistent with the region where neuronal cells were most affected metabolically by kainic acid^44^. This difference suggests that tubulin in mossy fibres of the stratum lucidum is mainly converted to glutamylated Δ2-tubulin. Indeed, DTE41-stained substrates were colocalised with the mossy fibre marker, calbindin, and most strongly detected at the edge of calbindin signals, indicating that the increase of DTE41 signals occurred near axonal terminals close to synapses. The presence of presynaptic kainate receptors in mossy fibres supports this idea^45^. In addition, this is in contrast to DTE41 signals that were primarily weak in white matters where synapses are rare.

In AD, DTE41-detected signals were increased in 3 different regions of the hippocampus, the CA1, CA2, and CA3 (Fig. 5). Similar to kainic acid-induced acute epileptic seizure, the increase in DTE41 signals indicates an increase in glutamylated Δ2-tubulin, given that Δ2-tubulin levels were not changed in AD hippocampus. The possible increase in glutamylated Δ2-tubulin in the apical dendrites of pyramidal neurons is partially consistent with a previous study that showed that the relative level of glutamylated tubulin against α-tubulin is increased in the apical dendrites of AD brain^31^, although there are some differences between our results and findings shown in that study. In our experiments, the signal of α-tubulin (DM1A) was not significantly decreased. This might be due to the different antibodies used; albeit this cannot be fully argued because information about the antibody used in that study was not provided. Alternatively, disease stages used in that study might be later than in our samples, though Braak stage was not described^31^. Of interest, the DTE41-detected signal, i.e. possible glutamylated Δ2-tubulin, was increased in mossy fibres of the CA3 region in AD samples. This raises a question about the molecular mechanisms underlying the specific increase in glutamylated Δ2-tubulin in mossy fibres. One study provides a clue; impaired inhibitory circuit and spontaneous seizure epilepsy are observed in a mouse model of AD^46^. This fits well with our proposed model for the similar selective increase in glutamylated Δ2-tubulin in the mossy fibre-rich stratum lucidum under acute kainic acid-induced epileptic seizure. Overactivation of mossy fibres due to the failure of inhibitory inputs could increase glutamylation on Δ2-tubulin up to levels that are difficult to be detected by mAb GT335. In fact, glutamylation is increased by the suppression of inhibitory inputs^41^. Calcium dyshomeostasis is well documented as an early event in AD^47^. The increase in glutamylated Δ2-tubulin might be an early event in AD. This scenario fits well with our finding that the signal intensities of DTE41 were highest in the early stage of AD (Braak IV) (Fig. 6a). Dysregulation of α-tubulin modifications near axonal terminals and presynapse could affect intra-axonal trafficking at the terminal as well as synaptic structures and function^5^, which induces neuronal dysfunction and ultimately degeneration.

In conclusion, the detection pattern of our new antibody DTE41 indicates that two different PTMs, glutamylation and Δ2 form, coexist on a single tubulin molecule, and that the double-modified tubulin, i.e. glutamylated Δ2-tubulin is increased in both acute epileptic seizure and slow-developing AD. Our findings imply that the alteration of tubulin PTMs occur preferentially on already-modified tubulin, and indicate that the increase in glutamylated Δ2-tubulin is a common event in both acute and slow-developing neurodegeneration.

## Methods

### Ethical standards

All the experiments analysing specimens of post-mortem human brains were performed according to the protocols approved by the Ethics Committee at the Hamamatsu University School of Medicine and the Choju Medical Institute, Fukushimura Hospital. Informed consent was obtained from all patients. Patient information was anonymised, and data presented in this study do not identify participants. All the experiments using mice were conducted according to the protocols approved by the Institutional Animal Care and Use Committee at the Hamamatsu University School of Medicine.

### Antibodies

A mAb (clone DTE41) was generated against a branched peptide that was the C-terminal portion of Δ2-tubulin having branched di-glutamates (Kohjin Bio, Saitama, Japan). The antibody subclass was IgG1κ. Primary and deduced quaternary structures of DTE41 were analysed as described in supplementary information. The culture supernatant of the P3U1 hybridoma producing mAb DTE41 was used for the experiments. The other antibodies used were anti-glutamylated tubulin mAb GT335 (AdipoGen, San Diego, CA); anti-tyrosinated tubulin mAb TUB-1A2, anti-α-tubulin mAb DM1A, anti-MAP2 mAb HM-2, anti-MAP2 polyclonal antibody (pAb), and anti-FLAG mAb M2 (Sigma-Aldrich, St. Louis, MO); anti-detyrosinated tubulin pAb, anti-Δ2-tubulin pAb, and anti-GAPDH mAb 6C5 (Millipore, Billerica, MA); anti-calbindin pAb (#ab11426; Abcam, Cambridge, UK); anti-TTLL1 antibody (Ikegami PNAS 2010); anti-AGTPBP1 (Proteintech, Rosemont, IL); AlexaFluor-conjugated secondary antibodies for immunostaining (Thermo Fisher Scientific, Waltham, MA); and horseradish peroxidase-conjugated secondary antibodies for immunoblotting (Jackson ImmunoResearch Laboratories, West Grove, PA).

### AD brain and non-AD brain samples

Post-mortem hippocampus specimens were prepared from 8 AD patients and 9 non-AD subjects in the Choju Medical Institute as previously described^48^. The brain was removed at autopsy, weighed, cut mid-sagittally, and examined for vascular and the other macroscopically detectable lesions. Specimens for experiments were taken from the right hemisphere, or from the unaffected hemisphere as determined by computed tomography, and fixed in 4% paraformaldehyde (PFA) after dividing into several regions. The specimens were embedded in paraffin and processed into 5 μm sections for histological and immunohistochemical examination. For diagnosis, tissue sections were stained using hematoxylin-eosin and Klüver-Barerra staining methods. Gallyas-Braak staining^49^,^50^ was used to detect senile plaques (SPs), cerebral amyloid angiopathy (CAA) and neurofibrillary tangles (NFTs). Amyloid histopathology was assessed according to the Consortium for Establishing a Registry for Alzheimer’s Disease (CERAD) scores^51^ for SPs, and Braak staging for NFTs^52^ were quantified as indicative of AD pathology. The serial sections of specimens were also stained with mAb 6E10 (BioLegend, San Diego, CA) for detecting Aβ, mAb AT8 (Fujirebio Europe, Gent, Belgium) for phosphorylated paired helical filament (PHF)-tau (pSer202/pThe205), mAb pSyn#64 (Wako, Osaka, Japan) for phosphorylated α-synuclein, and mAb 11–9 (Cosmo Bio, Tokyo, Japan) for phosphorylated TAR DNA-binding protein 43 kDa (TDP-43) (phosphor Ser409/410), to exclude false positive diagnosis of AD.

### Kainic acid-induced epileptic seizure mouse model

Eight-week-old male mice were intraperitoneally injected with kainate (25 mg/kg body weight). Mice showing severe and conclusive seizure at 30 min after kainate injection were used. Mice injected with phosphate-buffered saline (PBS) were used as controls. Mice were fixed with 4% PFA through perfusion fixation at 6 h after the injection. Brains were further fixed in 4% PFA/PBS at 4 °C overnight after dissection. The fixed brains were incubated in 10% and 20% sucrose at 4 °C for 20 h and then embedded in OCT compound (Sakura Finetek, Tokyo, Japan) with metal attachment at −190 °C. The frozen OCT-embedded tissue blocks were sagittally sectioned at 10 μm in thickness using a cryostat (CM 3050; Leica, Wetzlar, Germany) at −20 °C. The sagittal brain sections were attached on MAS-coated glass slides (Matsunami, Osaka, Japan).

### Recombinant proteins and synthetic peptides

A complementary DNA (cDNA) fragment corresponding to amino acids 412–451 (GMEEGEFSEAREDMAALEKDYEEVGVDSVEGEGEEEGEEY) of human α-tubulin 1A was inserted into pMAL-c2x. To generate detyrosinated form and Δ2 form, the last codon (TAC for Tyr) or the penultimate codon (GAA for Glu) was replaced with a stop codon (TAG or TAA, respectively) using the Quick Change^®^ II Site-Directed Mutagenesis Kit (Agilent Technologies, Santa Clara, CA). The sequences were checked with the Prism 3130 sequencer (Applied Biosystems, Waltham, MA). The maltose binding protein-fused α-tubulin 1A tails were expressed in an *E. coli* strain, BL21-CodonPlus^®^-RIL Competent Cells (Agilent Technologies) by induction with a final concentration of 0.3 mM isopropyl-γ-thiogalactopyranoside (IPTG) at 37 °C. Harvested cells were sonicated in PBS containing a protease inhibitor cocktail (Roche, Basel, Switzerland) on ice using an ultrasonic processor (Microson^TM^ Ultrasonic Cell Disruptor XL; Misonix, Farmingdale, NY). The supernatants obtained after 20000 × *g* centrifugation were subjected to western blotting. The C-terminal peptides of Δ2-tubulin possessing different lengths of glutamates chains were purchased from Peptide, Inc. (Japan). The primary C-terminus of synthetic peptides was converted to –COOH, which was determined by mass spectrometry results provided by the manufacturer along with peptides.

### Enzyme-linked immunosorbent assay (ELISA)

Synthetic peptides were coupled to bovine serum albumin (BSA) using glutaraldehyde. BSA dissolved in glutaraldehyde without the addition of peptides was used as the negative control. Indirect ELISA was carried out in 96-well flat-bottom microtitre plates (F96 Maxisorp NUNC-Immuno plate; Thermo Fisher Scientific). The cross-linked peptides were adsorbed to the well surface of ELISA plate by overnight incubation at 4 °C. The wells were blocked with 10% goat serum for 2 h at room temperature. Serial dilutions of mAb DTE41 in 5% goat serum blocking buffer were added to the wells and incubated overnight at 4 °C. The primary antibody was labelled with HRP-conjugated anti-mouse IgG goat antibodies (1:10000) for 2 h at room temperature. The wells were filled with 100 μL of 3,3′,5,5′-Tetramethylbenzidine (TMB) substrate solution. After colour development, 100 μL of 1N hydrochloric acid was added to stop the reaction, and the absorbance of 450-nm light was recorded as optical density (OD) values with a microplate reader (Synergy^TM−^HT; Bio-TEK Instruments, Winooski, VT).

### Cell culture and plasmid transfection

Cells were cultured and transfected with plasmids as previously described^53^. Human embryonic kidney (HEK) 293 T cells and COS7 cells were grown in Dulbecco’s modified Eagle’s medium (DMEM) supplemented with 10% foetal bovine serum (FBS) at 37 °C with 5% CO_2_. IMCD3 cells were grown in DMEM/F12 medium supplemented with 10% FBS at 37 °C with 5% CO_2_. For plasmid transfection, the cells with 50–75% confluency were transfected by using Lipofectamine^TM^ 2000 (Thermo Fisher Scientific) or polyethylenimine (Polysciences, Inc., Warrington, PA). Transfected cells were subjected to experiments at 24 h after transfection. The plasmids used were pCMV-TTLL5–Flag, pEGFP-TTLL5, and pCMV-CCP1-Flag. The empty pCMV-Tag vector was used for mock transfection. The P3U1 hybridoma was grown in RPMI medium supplemented with 15% FBS and Hybridoma Fusion and Cloning Supplement (Roche). To prevent depolymerisation of the microtubules, the cells were treated with 1 μM taxol for 1 h prior to fixation.

### Immunoblotting

Mouse organs were homogenised in 1× sample buffer (Wako) by a Teflon homogeniser. HEK293T cells were lysed in a hypotonic lysis buffer (50 mM Tris, pH 7.6, 1% Triton X-100). The supernatants at 20000 × g were used for western blotting. Protein concentration was determined by Bradford’s method. Proteins separated in SDS-PAGE were transferred to polyvinylidene fluoride membrane (PVDF; GE Healthcare, Chicago, IL), and reacted with specific antibodies (DTE41 at 1:200, GT335, 1:5000; DM1A, 1:5000; Tub-1A2, 1:1000; anti-detyrosinated tubulin pAb, 1:500; anti-Δ2-tubulin pAb, 1:1000; M2, 1:5000; 5C6, 1:3000; anti-TTLL1 pAb, 1:5000; anti-CCP1 pAb, 1:2000). The membranes were incubated with HRP-labelled secondary antibodies (1:10000) and were developed with enhanced chemiluminescence reagent (ECL; GE Healthcare), and images were captured with a CCD camera (LAS-3000 mini; Fujifilm, Tokyo, Japan).

### Immunocytochemistry

Immunocytochemistry was performed as previously described^54^. COS7 cells and IMCD3 cells were fixed with methanol at −20 °C for 20 min or with 4% paraformaldehyde at 37 °C for 30 min. The fixed cells were blocked with 5% goat serum and 0.1% Triton X-100 in PBS at room temperature for 1 h, and then labelled with primary antibodies (DTE41, 1:200; M2, 1:5000; GT335, 1:2000; anti-Δ2-tubulin pAb, 1:500; DM1A, 1:5000). The primary antibodies were labelled with the fluorescence-labelled secondary antibodies at 4 μg/mL. Zenon^®^ antibody labelling kits (Thermo Fisher Scientific) were used when co-staining with primary antibodies derived from the same host. The fluorescence signals were observed with confocal laser scanning microscope (Fluoview FV1000, Olympus, Tokyo, Japan; SP8, Leica).

### Immunohistochemistry

Mouse brain sections were blocked with 5% goat serum containing 0.1% Triton X-100 in PBS for 1 h at room temperature. The sections were incubated overnight at 4 °C with appropriate antibodies (DTE41, 1:100; GT335, 1:2000; HM-2, 1:1000; anti-Δ2-tubulin pAb, 1:1000; anti-MAP2 pAb, 1:1000; anti-calbindin pAb, 1:1000; DM1A, 1:2000). Primary antibodies were labelled with AlexaFluor-conjugated secondary antibodies at 50 μg/mL for 1 h at room temperature. To confirm that mAb DTE41 binds specifically to the antigen of interest, the primary antibody was pre-incubated with antigen peptides at 10:1 molar ratio of antigen and antibody at 4 °C overnight. To guarantee that mAb DTE41 signals were bona fide, tissue sections were stained with secondary antibodies without adding the primary antibody. The fluorescent signals were observed using confocal laser scanning microscope (Fluoview FV1000, Olympus, SP8, Leica).

The sections of AD and non-AD samples were deparaffinised in xylene and rehydrated in a graded ethanol series. Heat-induced antigen retrieval was performed in sodium citrate buffer (pH 6.0) at 95 °C for 20 min. Endogenous peroxidase was inactivated by treating tissue sections with 3% H_2_O_2_/methanol for 15 min. The sections were blocked with 5% goat serum containing 0.1% Triton X-100 in PBS at room temperature for 1 h, and then labelled with primary antibodies (DTE41 at 1:100; GT335 at 1:2000; DM1A at 1:2000; anti-Δ2 tubulin pAb at 1:500) at 4 °C overnight. The primary antibodies were labelled with HRP-conjugated secondary antibodies (1:200, Vector Laboratories, Burlingame, CA) for 1 h at room temperature. The signal was visualised by using a DAB substrate kit (Vector Laboratories). Stained sections were dehydrated, and mounted with Harleco Synthetic Resin mounting medium (Sysmex, Kobe, Japan). Images were taken with Olympus BX51 microscope equipped with Olympus DP-72 camera.

### Quantification and statistical analysis

ImageJ (http://rsb.info.nih.gov/ij/index.html) was used to quantify signal intensities. Band intensities of immunoblot were measured with a built-in command. Immunofluorescence signals were extracted as greyscale. Mean signals in the area without tissue sections were considered as background. The background signal was subtracted from the mean signal in the region of interest (ROI). Photographs of DAB-stained slides were converted to 16-bit greyscale images. The darkness in the greyscale was measured as signals in the ROI. Statistical significances were evaluated with two-tailed Student’s *t*-test. A Pearson’s test was performed to analyse the correlations between immunochemical signals and Braak stages, disease duration, age at death, or post-mortem intervals. p < 0.05 was considered as statistically significant. Quantified data were shown as mean ± standard error of mean (S.E.M), where the number of animals used, experiments done, or samples were provided as n or N.

## Additional Information

**How to cite this article**: Vu, H. T. *et al*. Increase in α-tubulin modifications in the neuronal processes of hippocampal neurons in both kainic acid-induced epileptic seizure and Alzheimer’s disease. *Sci. Rep.* **7**, 40205; doi: 10.1038/srep40205 (2017).

**Publisher's note:** Springer Nature remains neutral with regard to jurisdictional claims in published maps and institutional affiliations.

## Supplementary Material

Supplementary Information

## Figures and Tables

**Table 1:** Demographic data for patients with Alzheimer’s disease (AD) and non-AD subjects.

No	Diagnosis	Gender	Age at death (years)	Duration of disease (years)	Braak stage	CERAD score	Postmorterm interval (hours)	Clinical diagnosis
1	AD	Female	82	12	V	C	18	Alzheimer’s disease, chronic subdural hematoma, lung cancer
2		Female	90	6	IV	C	6	Alzheimer’s disease, lung cancer
3		Female	82	6	VI	C	6	Alzheimer’s disease
4		Female	88	16	VI	C	6.5	Alzheimer’s disease
5		Female	74	18	VI	C	17	Alzheimer’s disease
6		Male	93	6	IV	C	3	Alzheimer’s disease, dementia with Lewy Bodies
7		Female	88	10	IV	C	5	Alzheimer’s disease, senile dementia with neurofibrillary tangle
8		Male	76	1	IV	C	10	Alzheimer’s disease, pneumonia, pancytopenia, kidney failure
9	non-AD	Male	72	2	I	0	12.5	Lung cancer brain metastasis
10		Female	80	12	II	0	3	Frontotemporal lobar degeneration
11		Male	98	2	I	C	4.5	Dementia, pneumonia
12		Male	85	1	I	A	2.5	Dementia, duodenal ulcer
13		Male	76	0.5	I	0	3.5	Cerebral infarction, pneumonia
14		Female	85	4	II	B	2.5	Subarachnoid hemorrhage, acute myocardial infarction
15		Male	65	5	I	0	14	Dementia, pancreatic cancer
16		Male	83	12	I	0	14	Dementia, cerebral infarction, normal pressure hydrocephalus
17		Male	78	9	I	B	3	Vascular dementia
