# Supplementary material for: Increase in α-tubulin modifications in the neuronal processes of hippocampal neurons in both kainic acid-induced epileptic seizure and Alzheimer’s disease
Source: Sci Rep. 2017 Jan 9;7:40205. doi: 10.1038/srep40205 (PMC5220350; doi:10.1038/srep40205)
Supplement: Supplementary Information [file srep40205-s1.pdf]

SUPPLEMENTARY INFORMATION

Increase in α-tubulin modifications in the neuronal processes of hippocampal neurons in both kainic acid-induced epileptic seizure and Alzheimer's disease

Hang Thi Vu[1], Hiroyasu Akatsu[2,3], Yoshio Hashizume[2], Mitsutoshi Setou[1,4,5,6,7,]*, Koji Ikegami[1,]*

[1] Department of Cellular and Molecular Anatomy, and International Mass Imaging Center, Hamamatsu University School of Medicine, Hamamatsu, Japan

[2] Choju Medical Institute, Fukushimura Hospital, Toyohashi, Japan

[3] Department of Medicine for Aging in Place and Community-Based Medical Education, Nagoya City University Graduate School of Medical Sciences, Nagoya, Japan

[4] Department of Systems Molecular Anatomy, Preeminent Medical Photonics Education and Research Center, Hamamatsu University School of Medicine, Hamamatsu, Japan

[5] Department of Anatomy, The University of Hong Kong, Hong Kong, China

[6] Division of Neural Systematics, National Institute for Physiological Sciences, Okazaki, Japan

[7] Riken Center for Molecular Imaging Science, Kobe, Japan

* Address correspondence to: Mitsutoshi Setou (setou@hama-med.ac.jp) or Koji Ikegami (kikegami@hama-med.ac.jp)

SUPPLEMENTARY METHODS

Cloning and sequencing of antibody variable regions

mRNA was extracted from the cultured P3U1 hybridoma producing mAb DTE41 with Sepasol RNA I (Nacali Tesque, Kyoto, Japan). cDNA was generated with total RNA by using the Superscript III First-Strand Synthesis Kit for RT-PCR (Invitrogen). PCR was performed by using the following primers: for the variable region of γ1 heavy chain (Forward: 5'-atgRaSttSKggYtMaRctKgRtt; Reverse: 5' ATAGACAGATGGGGGTGTCGTTTTGGC); for the variable region of κ light chain (Forward: 5'-ATGGATTTTCAAGTGCAGATTTTCAG; Reverse: 5'-GGATACAGTTGGTGCAGCATC). In the forward degenerate primer, atypical symbols indicate the following: R = A or G; S = G or C; K = G or T; Y = C or T; M = A or C. After cloning the fragment into pCR-TOPO-blunt II vector (Invitrogen), the sequence was checked with the Prism 3130 sequencer (Applied Biosystems).

Prediction of molecular structure of antibody variable region

The Prediction of ImmunoGlobulin Structure (PIGS) website was used to get a template-based structural prediction of the antibody variable region[1]. After the first prediction was obtained, the structure was subjected to structural refinement with a classical algorithm, ClassicRelax, mounted in PyRosetta[2,3].

Immunoprecipitation and two-dimensional electrophoresis

HEK293T cells were transfected with TTLL5-flag alone or co-transfected with TTLL5-flag and CCP1-flag. Cells expressing TTLL5 only were lysed with a lysis buffer:

50 mM MES-NaOH, pH 7.2, 1% Triton X-100, 100 mM NaCl, 1 mM EDTA, and protease inhibitor cocktail (Roche). Cells expressing both TTLL5 and CCP1 were lysed with buffer for isoelectric point focusing (IEF) as described previously[4]. Tyrosinated α-tubulin (Tyr-tubulin) was purified from cell lysates expressing TTLL5 alone with mAb 1A2 (Sigma) and protein-G sepharose (GE Healthcare). Immunoprecipitated Tyr-tubulin was extracted with IEF buffer as described previously[4]. Two-dimensional electrophoresis was performed using Immobiline DryStrip of 7 cm in length and pH range 3 to 5.6, and Maltiphor II (GE Healthcare) as described previously[4]. Separated proteins were transferred onto PVDF membrane, and detected by western blot.

SUPPLEMENTARY REFERENCES

1. Marcatili P, Rosi A, Tramontano A (2008) PIGS: automatic prediction of antibody structures. Bioinformatics 24: 1953-1954.
2. Chaudhury S, Lyskov S, Gray JJ (2010) PyRosetta: a script-based interface for implementing molecular modeling algorithms using Rosetta. Bioinformatics 26: 689-691.
3. Bradley P, Misura KM, Baker D (2005) Toward high-resolution de novo structure prediction for small proteins. Science 309: 1868-1871.
4. Ikegami K, Horigome D, Mukai M, Livnat I, MacGregor GR, Setou M (2008) TTLL10 is a protein polyglycylase that can modify nucleosome assembly protein 1. FEBS Lett. 582: 1129-1134.

SUPPLEMENTARY FIGURE LEGENDS

Figure S1. Primary and deduced quaternary structures of the variable region of mAb DTE41. (**a**, **b**) Nucleotide sequences and deduced amino acid sequences of the heavy chain (**a**) and light chain (**b**) of the DTE41 variable region. The data lack leader sequences. CDRs, complementarity-determining regions, were defined according to the criteria of Kabat and Chotia. Basic amino acids in CDRs are highlighted in red. (**c**) The deduced quaternary structure of the DTE41 variable region. The DTE41 variable region has 9 basic amino acids (magenta). Cyan, CDRs in HC. Light green, CDRs in LC. Blue, nitrogen atoms in side chains of basic amino acids in CDRs.

Figure S2. Specificity of mAb DTE41. (**a**) ELISA of mAb DTE41 and anti-Δ2-tubulin pAb against peptides. Antibody dilution rates were 1:200 for mAb DTE41 and 1:1,000 for anti-Δ2-tubulin pAb. (**b**) Evaluation of mAb DTE41 affinity on glutamylated Tyr-tubulin. mAb DTE41 did not recognise glutamylated Tyr-tubulin purified from TTLL5-overexpressing HEK293T cells with mAb 1A2 (right, top). Tyr-tubulin was detected with mAb 1A2 (middle row). Glutamylation was detected with mAb GT335 (bottom row). (**c**) Co-labelling IMCD3 cells with mAb DTE41 and anti-Δ2-tubulin pAb. mAb DTE41 labelled a portion of Δ2-tubulin-positive cells.

Figure S3. Confirmation of specificities of mAb DTE41-detected signals on mouse brain sections. (**a**) Immunohistochemical staining of whole mouse brain section by omitting mAb DTE41. Green, background signals of anti-mouse secondary antibodies (AlexaFluor 488); red, MAP2; blue, nuclei, Scale bar, 2 mm. (**b**) Immunohistochemistry

with mAb DTE41 (top), pre-depleted mAb DTE41 with antigen peptides (Δ2 + 4E) (middle), and primary antibody omission (bottom). Scale bars, 2 mm.

Figure S4. Co-staining of mouse brain section with mAb DTE41 and anti-Δ2-tubulin pAb. (**a**) Immunohistochemical staining of whole mouse brain section with mAb DTE41 and anti-Δ2-tubulin pAb. Green, mAb DTE41; red, Δ2-tubulin; blue, nuclei. Scale bar, 2 mm. (**b**, **c**, **d**) Magnified images of the cerebral cortex (**b**), cerebellum (**c**), and hippocampus (**d**) with mAb DTE41 and anti-Δ2-tubulin pAb. Green, mAb DTE41; red, Δ2-tubulin; blue, nuclei. Scale bars, 50 μm.

Figure S5. Verification of specificity of mAb DTE41-detected signals in kainate-treated hippocampus. (**a**) Immunohistochemical staining of kainate-administrated the hippocampal CA3 region with anti-calbindin pAb by omitting mAb DTE41. Green, background signals of anti-mouse secondary antibodies; red, calbindin; blue, nuclei. Scale bar, 50 μm. (**b**) Immunohistochemical staining of kainate-treated or non-treated control rat hippocampal CA3 region (Upper row). The bottom row is negative control showing no background staining with anti-mouse secondary antibodies. Scale bars, 150 μm. (**c**) Quantitative analyses of immunofluorescence intensities in panel **b**.

Figure S6. Full-length blots. (**a**) is for Fig. 1c, (**b**) is for Fig. 2a, and (**c**) is for Fig. 3a.

Figure S7. Full-length blots. (**a**) is for Fig. 4a ,and (**b**) is for Fig. 4g

Figure S1

(a)

1- GAG GTG CAG CTT GTT GAG ACT GGT GGA GGA TTG GTG CAG CCT AAT GGG TCA TTG AAA CTC
E V Q L V E T G G G L V Q P N G S L K L

CDR1

61- TCA TGT GCA GCC TCT GGA TTC ACC TTC AAT ATC AAT GTC ATG AAT TGG GTC CGC CAG GCT
S C A A S G F T F N I N V M N W V R Q A

CDR2

121- CCA GGA AAG GGT TTG GAA TGG GTT GCT CGC ATA AGA AGT AAA AGT AAT AAT TTT GCA ACA
P G K G L E W V A **R** I **R** S **K** S N N F A T

181- TAT TAT GCC GAT TCA GTG AAA GAC AGG TTC ACC ATC TCC AGA GAT GAT TCA CAA AGC ATG
Y Y A D S V K D R F T I S R D D S Q S M

241- CTC TAT CTG CAA ATG AAC GAC TTG AAA ACT GAG GAC ACA GCC ATG TAT TAC TGT GTG AGG
L Y L Q M N D L K T E D T A M Y Y C V R

CDR3

301- TTT GGT AAG GGG GAT GCT ATG GAC TAC TGG GGT CAA GGA ACT TCA GTC ACC GTC TCC TCA
F G **K** G D A M D Y W G Q G T S V T V S S

(b)

1- CAA ATT GTT CTC ACC CAG TCT CCA GCA ATC ATG TCT GCA TCT CCA GGG GAG AAG GTC ACC
Q I V L T Q S P A I M S A S P G E K V T

CDR1

61- ATG ACC TGC AGT ACC AGC TCA AGT ATA AAT TAC ATG CAC TGG TAC CAG CAG AAG CCA GGC
M T C S T S S S I N Y M **H** W Y Q Q K P G

CDR2

121- ACC TCC CCC AAA AGA TGG ATT TAT GAC ACA TCC AAA CTG GCT TCT GGA GTC CCT GCT CGC
T S P K R W I Y D T S **K** L A S G V P A R

181- TTC AGT GGC AGT GGG TCT GGG ACC TCT TAT TCT CTC ACA ATC AGC AGC ATG GAG GCT GAA
F S G S G S G T S Y S L T I S S M E A E

CDR3

241- GAT GCT GCC ACT TAT TAT TGC CAT CAG CGG AGC AGT TAC CCA CGC ACG TTC GGT GCT GGG
D A A T Y Y C **H** Q **R** S S Y P **R** T F G A G

301- ACC AAG CTG GAG CTG AAA CGG
T K L E L K R

(c)

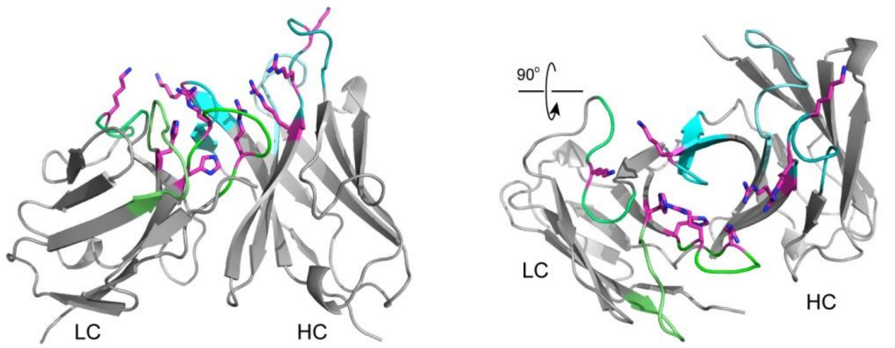

Figure S2

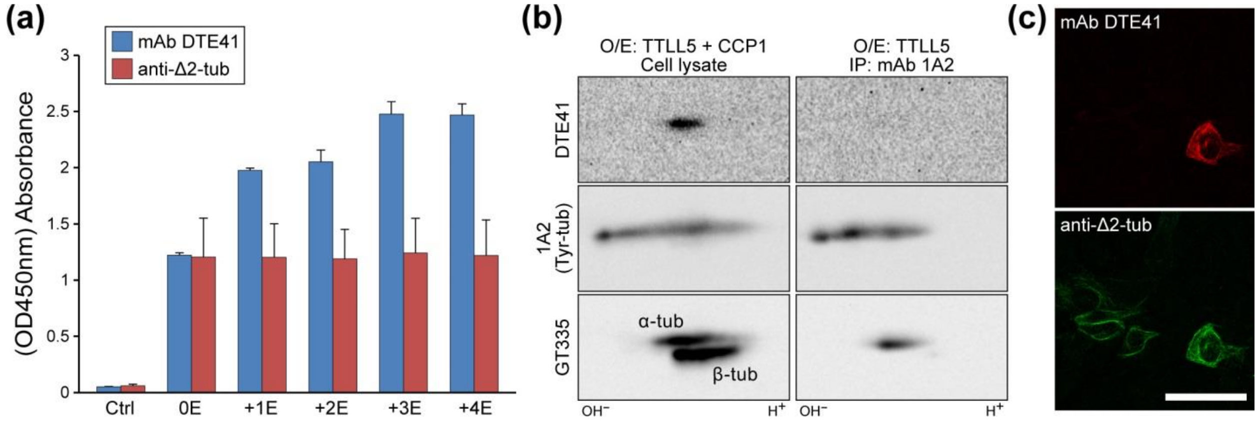
(a)
mAb DTE41
anti-Δ2-tub
(OD450nm) Absorbance
3
2.5
2
1.5
1
0.5
0
Ctrl
0E
+1E
+2E
+3E
+4E
(b)
O/E: TTLL5 + CCP1
Cell lysate
O/E: TTLL5
IP: mAb 1A2
DTE41
1A2
(Tyr-tub)
GT335
α-tub
β-tub
OH⁻
H⁺
OH⁻
H⁺
(c)
mAb DTE41
anti-Δ2-tub

Figure S3

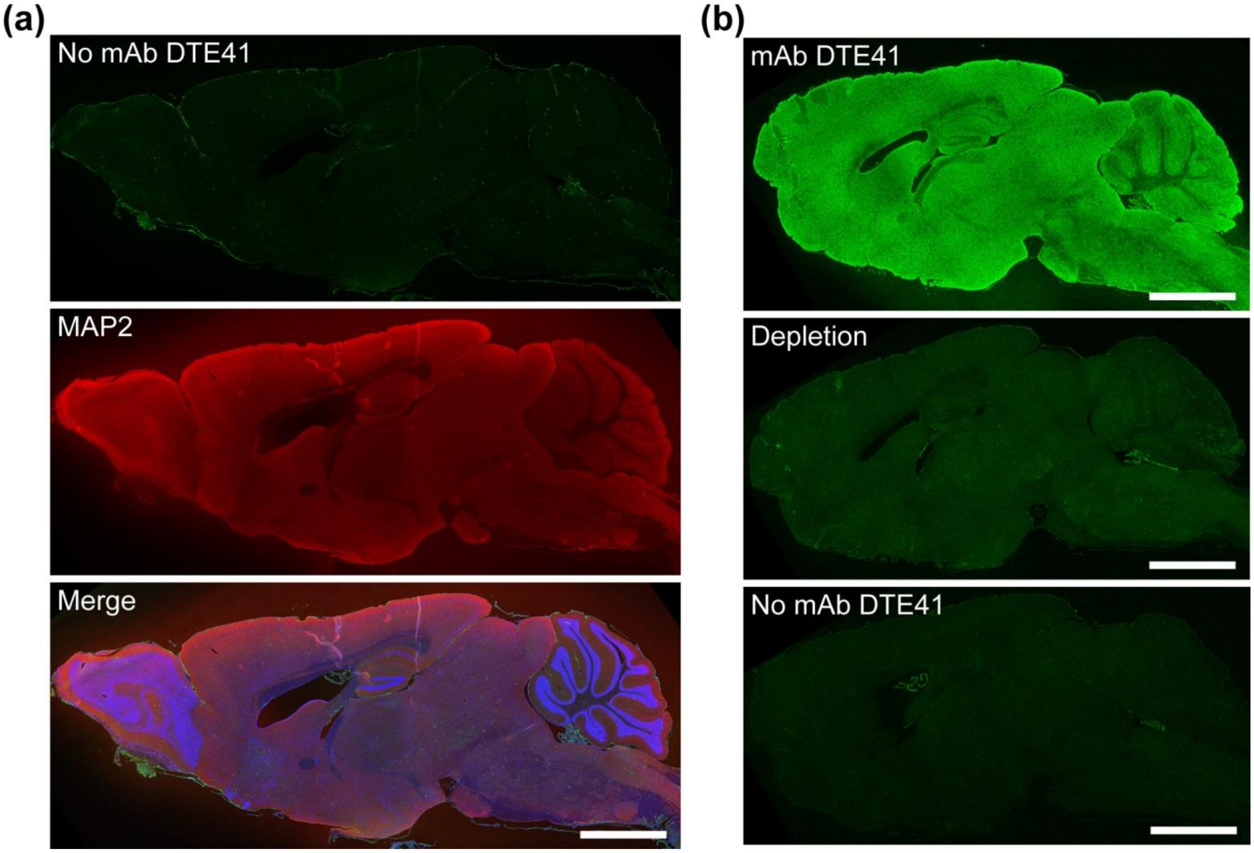

Figure S4

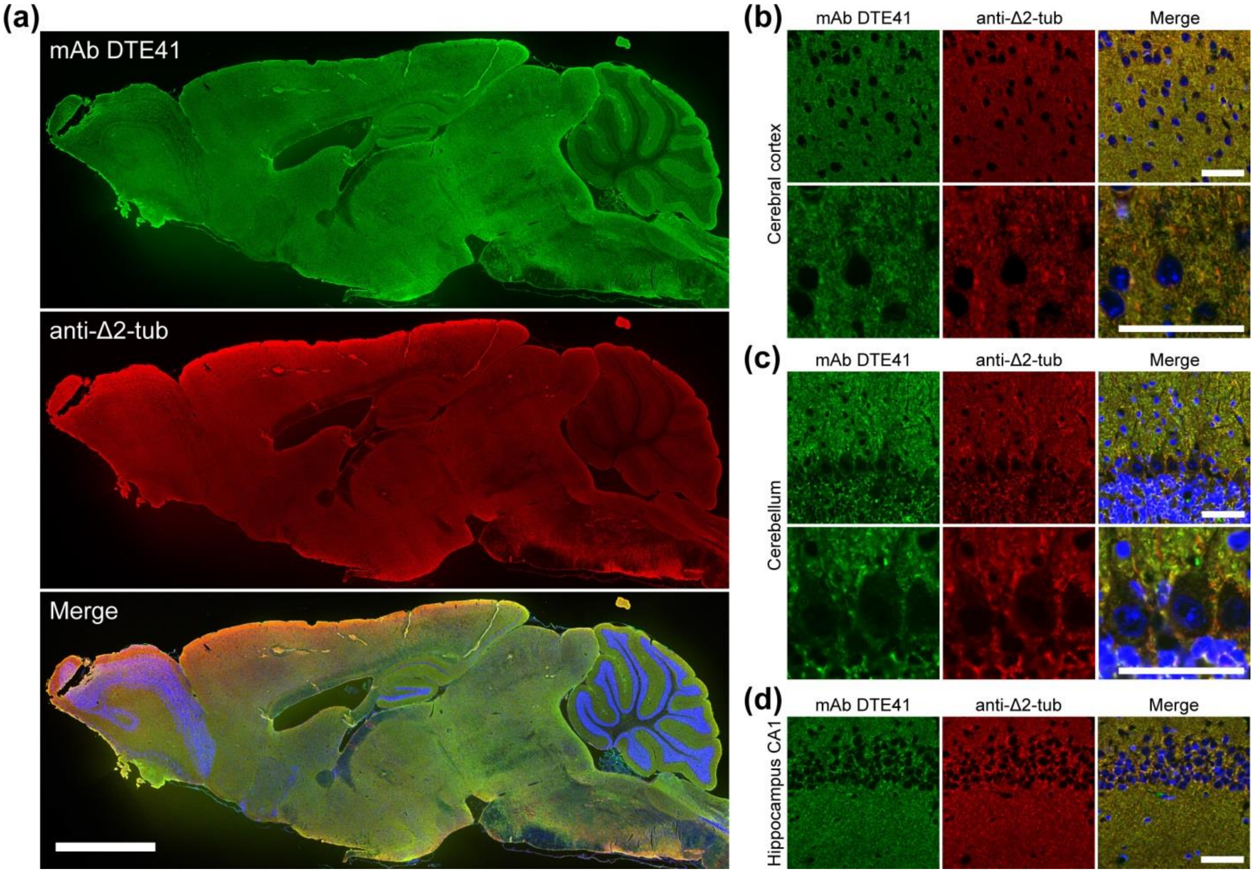

(a)
mAb DTE41
anti-Δ2-tub
Merge
(b)
mAb DTE41
anti-Δ2-tub
Merge
Cerebral cortex
(c)
mAb DTE41
anti-Δ2-tub
Merge
Cerebellum
(d)
mAb DTE41
anti-Δ2-tub
Merge
Hippocampus CA1

Figure S5

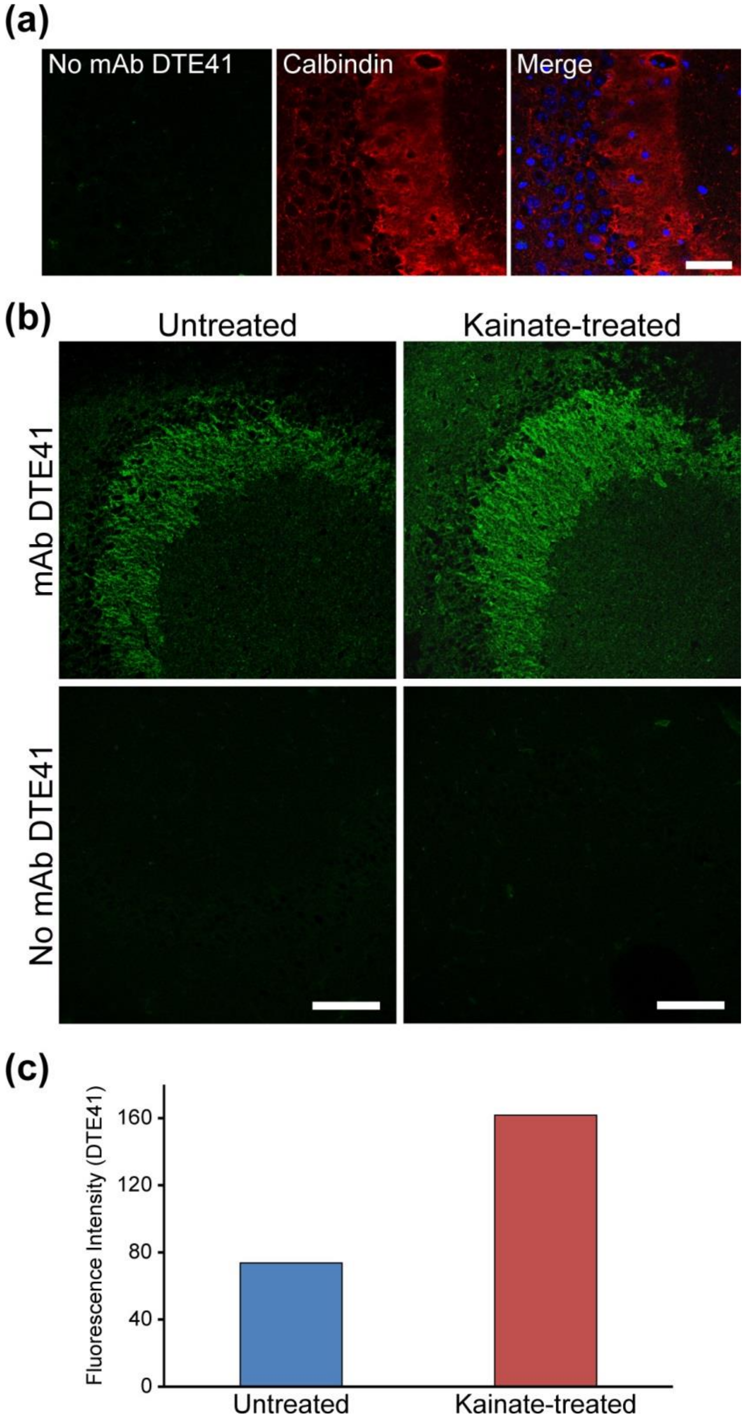

(a)
No mAb DTE41
Calbindin
Merge
(b)
Untreated
Kainate-treated
mAb DTE41
No mAb DTE41
(c)
Fluorescence Intensity (DTE41)
160
120
80
40
0
Untreated
Kainate-treated

Figure S6

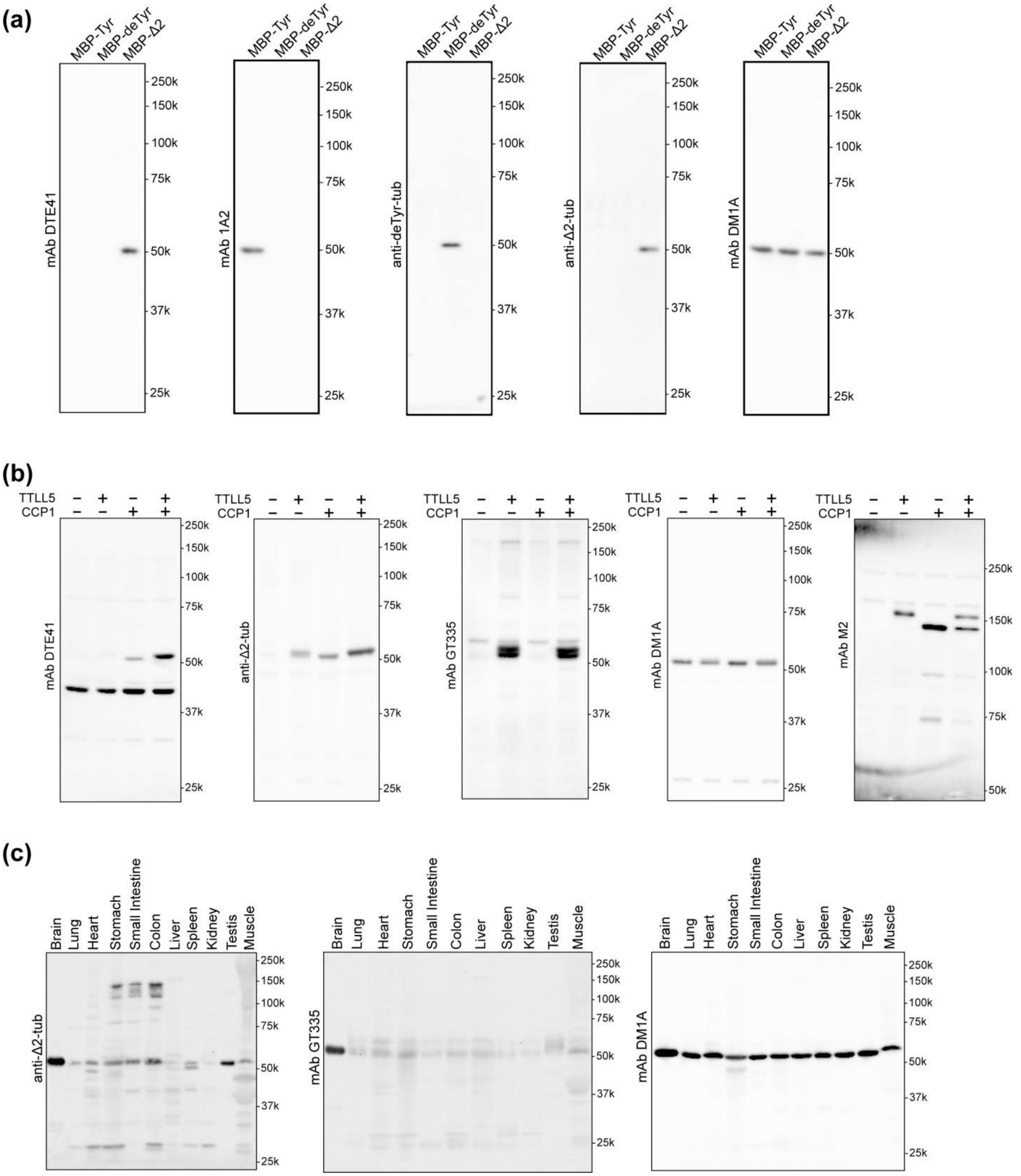
(a)
MBP-Tyr
MBP-deTyr
MBP-Δ2
mAb DTE41
mAb 1A2
anti-deTyr-tub
anti-Δ2-tub
mAb DM1A
250k
150k
100k
75k
50k
37k
25k
(b)
TTLL5
CCP1
mAb DTE41
anti-Δ2-tub
mAb GT335
mAb DM1A
mAb M2
(c)
Brain
Lung
Heart
Stomach
Small Intestine
Colon
Liver
Spleen
Kidney
Testis
Muscle
anti-Δ2-tub
mAb GT335
mAb DM1A

Figure S7

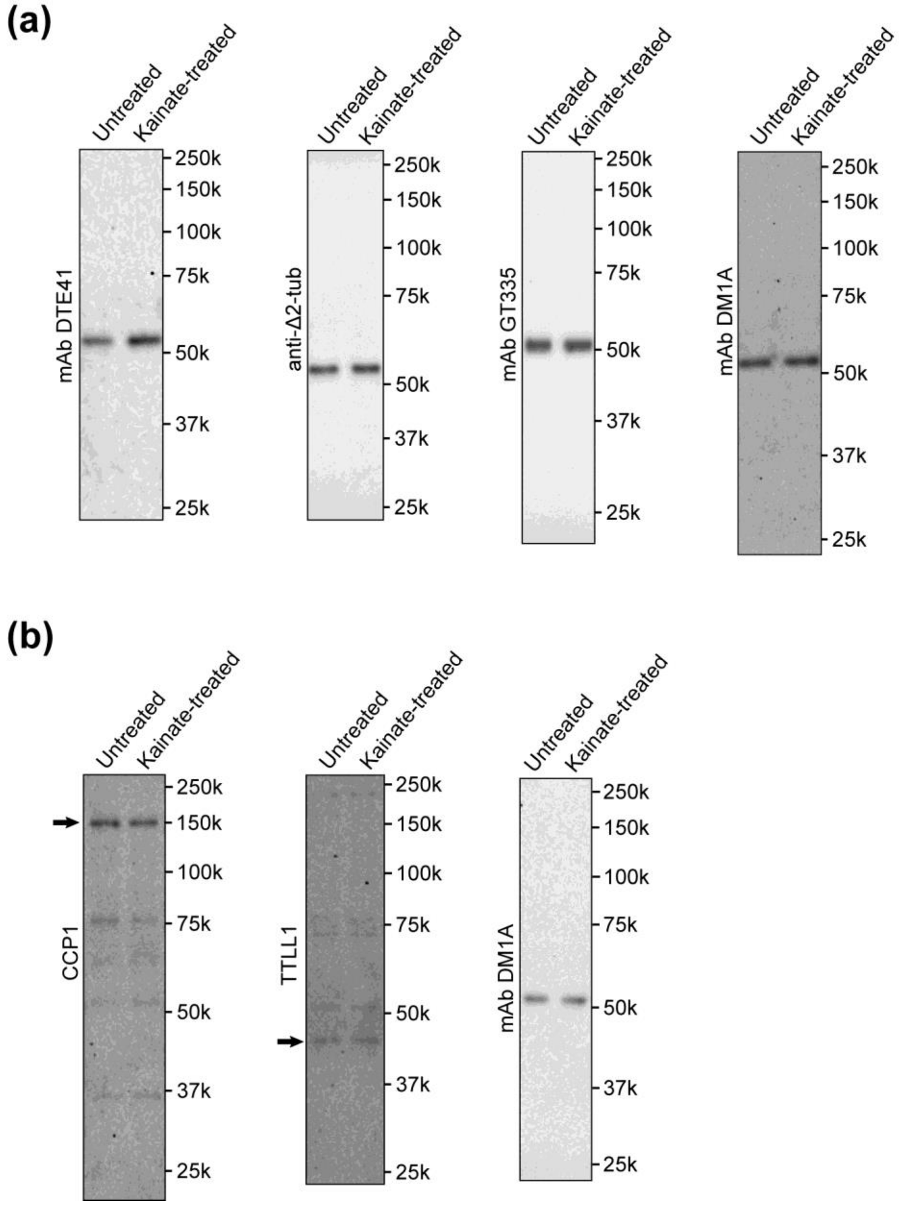
(a)
Untreated
Kainate-treated
mAb DTE41
anti-Δ2-tub
mAb GT335
mAb DM1A
250k
150k
100k
75k
50k
37k
25k
(b)
CCP1
TTLL1
mAb DM1A
